# Web Wrinkle Defects due to Temperature Profile in Roll-to-Roll Manufacturing Systems

**DOI:** 10.3390/polym15020457

**Published:** 2023-01-15

**Authors:** Jaehyun Noh, Minho Jo, Hojin Jeon, Minjae Kim, Jeongdai Jo, Changwoo Lee

**Affiliations:** 1 Department of Mechanical Design and Production Engineering, Konkuk University, 120 Neungdong-ro, Gwangjin-gu, Seoul 05029, Republic of Korea; 2 Department of Printed Electronics, Korea Institute of Machinery and Materials, 156, Gajeongbuk-ro, Yuseong-gu, Daejeon 34103, Republic of Korea; 3 Department of Mechanical Engineering, Konkuk University, 120 Neungdong-ro, Gwangjin-gu, Seoul 05029, Republic of Korea

**Keywords:** roll-to-roll manufacturing process, strain deviation, web handling, web temperature distribution, wrinkles

## Abstract

The roll-to-roll manufacturing system is extensively used for mass producing products made of plastic, paper, and fabric in several traditional industries. When flexible substrates, also known as webs, are heated and transported inside the dryer, an inconsistent temperature distribution occurs on the material in the machine direction (MD) and cross-machine direction (CMD). If rollers are not aligned in parallel on the same plane in the roll-to-roll web handling process, or if roller misalignment exists, strain deviation occurs in the web, resulting in lateral displacement and web wrinkles. Therefore, this study examined a wrinkle, which is a thermal deformation that occurs when an inconsistent web temperature distribution is formed on the material inside a dryer. The changes in the elastic modulus and thermal expansion of the web were also examined. Experiments were conducted using a PET film, and its elastic modulus and thermal expansion were examined. The results showed that the presence of a web wrinkle defect can cause a thickness deviation in the functional layer manufactured on the web. Moreover, an appropriate operating speed should be set to reduce the CMD temperature deviation, thereby reducing instances of wrinkle defects.

## 1. Introduction

The roll-to-roll manufacturing system is a continuous manufacturing process, in which high-speed manufacturing enables mass production [1,2,3]. Traditionally, roll-to-roll manufacturing is applied to manufacture products made from plastic film, paper, and fabric [4,5,6,7,8]. In recent years, the system has become a promising candidate for mass producing flexible batteries, flexible copper-clad layers, and bioelectric sensors on a thin substrate [9,10,11,12,13]. Flexible substrates typically used in the roll-to-roll manufacturing system are called webs, and the major process parameters are web speed and web tension [14,15,16,17]. Increasing the operating speed has been attempted to maximize productivity, but it can cause lateral displacement due to slippages between the material and roll and defective drying of the coating or printing layers [18,19,20,21,22]. Furthermore, unexpected deformation such as web wrinkles may occur on the material due to tension errors applied to the web [23,24,25,26]. Thus, optimizing the operating speed and tension in advance is critical because products must be discarded if such errors occur. Generally, the roll-to-roll manufacturing system consists of unwinding, infeeding, printing or coating, drying, outfeeding, and rewinding sections. The manufacturing process begins in the unwinding section, where the material is unwound, and the coating or printing process and drying process occur between the infeeding and outfeeding sections. After the functional layer is manufactured, batches of product are wound in a roll in the rewinding section for compact storing. In this process, many webs are wound on one central core in the final product, thereby increasing productivity; however, the quality of the functional layer may be degraded if excessive radial stress is generated within the wound roll. Noh et al. observed that radial stress increased over time due to residual stress and reported that the storage time of a wound roll needs to be minimized [27]. In particular, when the electrolyte layer manufactured by Roll-to-Roll slot die coating is stored in a compact wound roll form, it is confirmed that the thickness of the electrolyte layer decreases due to the excessive stress in a radial and hoop direction generated inside the wound roll as a large amount of web is wound. Open circuit voltage of SOFCs manufactured with such defects was measured and it was confirmed that excessive internal stress inside the wound roll could lead to poor electrical performance.

The web is heated inside a dryer to evaporate the solvent applied on the web during the process of manufacturing the functional layer. Various types of heat sources are applied, such as infrared lamps and hot air [28]. Due to the nature of the roll-to-roll system, the material is continuously transported and has tension applied due to the velocity difference of driven rolls on both sides, and thermal deformation occurs in the tensioned web inside the high-temperature dryer system [29,30,31]. Tension disturbance occurs in webs in the dryer system due to such deformation, which may cause register error, buckling, and wrinkles [32,33]. In addition, Feng et al. reported that web wrinkles may occur when tension across the width direction is non-uniform during the process of manufacturing printed electronics and proposed a method for checking the average web tension and tension variation to reduce these defects [34]. Lee et al. reported that web wrinkle defects and telescoping in a wound roll occur when a lateral motion error is generated in the roll-to-roll web handling process [35]. The web with lateral displacement partially slips where it contacts a roller, and therefore part of the web is deformed in the z-direction (ZD). If rollers are not aligned in parallel on the same plane (roller misalignment) during the roll-to-roll web handling process, strain deviation occurs in the web, which leads to lateral displacement and web wrinkles [36,37].

Furthermore, we examined the possibility of other causes of wrinkles that may occur on a web inside a dryer and conducted research accordingly. In general, temperature deviation occurs in the machine direction (MD) and in the cross-machine direction (CMD) of the web in the dryer when manufacturing the coated or printed layers through the roll-to-roll manufacturing process. This temperature deviation induces an elastic modulus and coefficient of thermal expansion, which are mechanical properties of the web that ultimately cause strain deviation. Large strain deviation in the web causes deformation in the ZD, which may generate visually observable wrinkles. When wrinkles are generated in the web, these wrinkles cause a thickness deviation in the machine direction (MD) and CMD during the coating or printing processes of the manufactured functional layer, which ultimately leads to degradation in the final products [38,39].

## 2. Materials and Methods

### 2.1. Roll-to-Roll Manufacturing Systems with Drying Section

**Figure 1:**
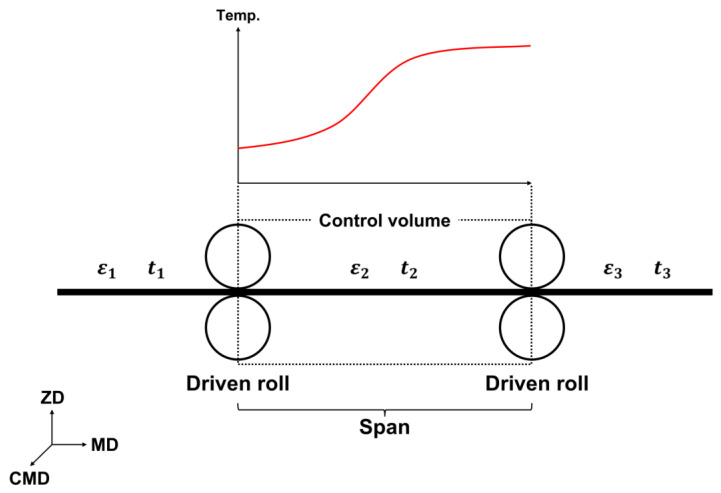
Schematic of span with web temperature variations. Note: ZD, z-direction; MD, machine direction; CMD, cross-machine direction, $\varepsilon_{1}$, $\varepsilon_{2}$, $\varepsilon_{3}$, strain of web at each span; $t_{1}$, $t_{2}$, $t_{3}$, tension of web at each span.

Shin et al. developed a mathematical model that explains the web tension behavior in the open loop section based on the law of conservation of mass, which states that the mass of a material transported within a controlled volume is constant [40]. As shown in Figure 1, Lee et al. further developed the mathematical model proposed by Shin et al. to explain the tension behavior reflecting the thermal effects of a web under high temperatures through a computer simulation and experimental studies [41]. Lee et al. expressed the thermal strain resulting from temperature changes, which was not considered in previous studies, and mitigated the limitations of previous studies by introducing the strain rate while considering the elastic modulus and coefficient of thermal expansion of the web, which vary under high-temperature conditions. The equivalent web strain within the span expressed by Lee et al. (Equation (2)) can be expressed as follows:

(1) $$\varepsilon_{eq}\left(t\right)=\frac{1}{L}\int_{0}^{L}\varepsilon \left(x,t\right)dx$$

where ε(x,t) is the strain of an infinitesimal element and L is the span length.

(2) $$\varepsilon_{eq}\left(t\right)=\varepsilon_{eq}^{e}\left(t\right)+\varepsilon_{eq}^{th}=\frac{t\left(t\right)}{AE_{eq}}+\frac{1}{L}\int_{0}^{L}\alpha \left(x\right)\left(\theta \left(x\right)-\theta_{1}\right)dx,$$

where $\varepsilon_{eq}^{e}\left(t\right)$, $\varepsilon_{eq}^{th}$, A, and $E_{eq}$ are the equivalent elastic strain, equivalent thermal strain, cross-sectional area of web, and equivalent elastic modulus, respectively. Furthermore, t(t), α, and θ are the tension of web, coefficient of thermal expansion of the web, and temperature.

(3) $$E_{eq}=\frac{L}{\int_{0}^{L}\frac{1}{E\left(x\right)}dx}$$

(4) $$\frac{d}{dt}T_{2}\left(t\right)=\frac{v_{10}E_{2eq}}{LE_{\theta 1}}T_{1}\left(t\right)-\frac{v_{20}}{L}T_{2}\left(t\right)+\frac{AE_{2eq}}{L}\left(V_{2}\left(t\right)-V_{1}\left(t\right)\right)-\frac{AE_{2eq}\varepsilon_{2eq}^{th}}{L}v_{20}$$

where T, V, and v are the web tension change, speed change, and velocity of roller, respectively. Equation (4) is a mathematical model for the tension behavior in a single span of a transported web assuming high-temperature conditions.

The polymer-based webs mostly used in roll-to-roll manufacturing are generally very thin (approximately 0.1 mm or less) and long [42]. Therefore, the web may be vulnerable to deformation under high-speed or high-strength conditions, and deformation can be significant—particularly under high temperatures [43,44]. However, the necessity of applying a high operating speed is on the rise to increase productivity at industrial sites that utilize roll-to-roll manufacturing. Under high-speed conditions, the possibility of a slip occurring increases when the speed of the material and linear speed of the idle roll are unequal. If the web slips on the roll, the coated layer on the web becomes unusable due to scratch defects, and displacement occurs in the lateral direction of the material, which results in a wound roll product with telescoping occurring in the rewinding section (final process) [45,46,47]. Generally, the operating tension is set higher to reduce the occurrence of a slip when a high operating speed is applied, because the problems arising from a slip can be prevented by increasing the operating tension and ensuring a sufficient traction force. In particular, in the roll-to-roll gravure printing process, as process conditions such as printing conditions, tension, operating speed, and nip pressure are changed, the degree of defect or conductance of the fabricated pattern may be significantly changed [48]. Accordingly, it is essential to optimize main process conditions in the roll-to-roll manufacturing system. However, excessive deformation may occur in the material due to changes in the elastic modulus and coefficient of thermal expansion that are caused when the web is heated to a high temperature in the drying section [49,50].

**Figure 2:**
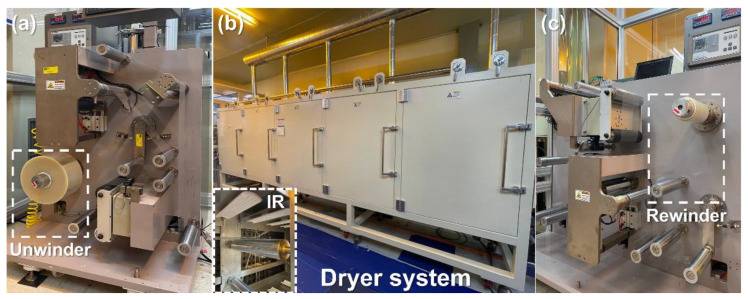
Roll-to-roll manufacturing system: (**a**) unwinding section; (**b**) dryer system with air and infrared (IR) heating system; (**c**) rewinding section.

An experiment was conducted to examine the actual behavior of a material under high-temperature conditions, as shown in Equation (4). In the roll-to-roll manufacturing system, which is installed at Konkuk University, Seoul, Republic of Korea, shown in Figure 2, a web transporting experiment was conducted for a polyethylene terephthalate (PET) film. In the unwinding section, the web is transported to the entire roll-to-roll manufacturing system, as shown in Figure 2a, as the wound roll is unwound. As shown in Figure 2b, the coating layer on the web can be dried with a drying system composed of hot air and infrared methods. After the manufacturing process, as shown in Figure 2c, the transported web is wound in a rewinder and compactly stored in a wound roll form. When the material was transported without operating the dryer in the pre-experiment, no deformation was observed in the web. However, when the dryer was set to 100 °C or higher in the main experiment, web wrinkles were observed in the transported tensioned web. Therefore, this study aimed to examine a wrinkle—a thermal deformation that occurs when inconsistent web temperature distribution is formed on the material inside a dryer—as well as changes in the elastic modulus and thermal expansion of the web.

### 2.2. Web Temperature and Strain Distribution

This study conducted an experiment using a PET film (CD901, Kolon Inc., Seoul, Republic of Korea) commonly used in roll-to-roll manufacturing. PET film is transparent, has great tensile strength, and has a high-temperature resistance, and it is therefore widely used. The properties of the PET film used in the experiment are presented in Table 1.

**Figure 3:**
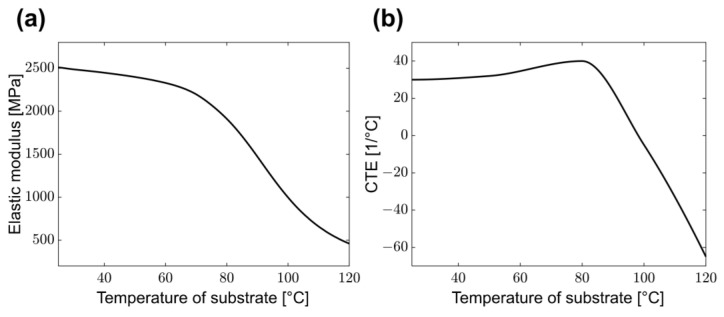
Mechanical and thermal properties of PET film by substrate temperature: (**a**) Elastic modulus; (**b**) coefficient of thermal expansion (CTE).

**Figure 4:**
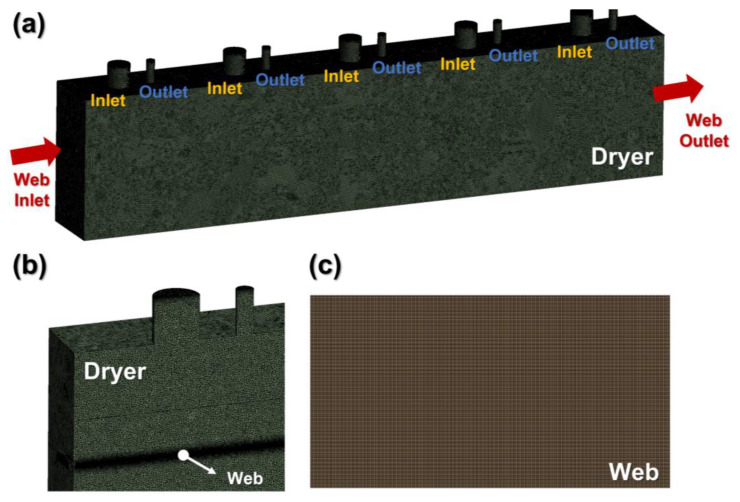
CFD mesh model: (**a**) roll-to-roll dryer system; (**b**) dryer cross-section; (**c**) solid model of the moving web.

In general, the elastic modulus and coefficient of thermal expansion of a PET film vary depending on the temperature. Figure 3a shows the elastic modulus of the PET film under different temperatures. To measure the elastic modulus, a tensile test was performed by heating a chamber from 25 °C to 120 °C. Figure 3b shows the coefficient of thermal expansion according to the temperature when the PET film was heated from room temperature to 120 °C. The fluid-governing equations were solved in ANSYS 2022 R1 Fluent (ANSYS Inc., Canonsburg, PA, USA). Within the CFD model, the effect of web transfer was considered by assigning a solid motion condition to the web. Figure 4a shows a mesh model throughout the 5.5-m-long dryer system. Table 2 presents the specifications of the dryer model examined in this study and the boundary conditions applied to the CFD model. Five fluid inlets and five outlets are installed at the top of the dryer, respectively. Figure 4b represents the cross-section of the dryer mesh model, and a solid moving web is located inside. Figure 4c indicates the mesh model of the web inside the dryer. Hot air and infrared were selected as heat sources to ensure that the web quickly converged to the set temperature. As shown in Table 3, a total of nine cases were devised to deduce the temperature deviation and strain on the material according to the operating speed and operating tension.

**Figure 5:**
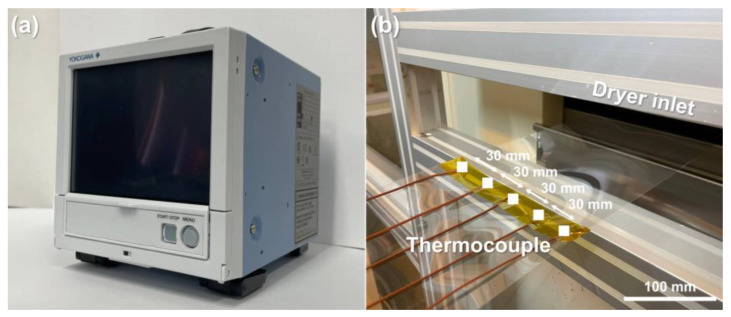
Web temperature measurement unit: (**a**) temperature data acquisition device; (**b**) thermocouples equally spaced in cross-machine direction.

The maximum operating speed was set to ensure that there was time to sufficiently heat the material, considering the total length of the dryer and drying conditions. The minimum operating speed was selected as the minimum value within a range where the tension could be properly controlled while considering the specifications of the roll-to-roll manufacturing system used in the experiment. The maximum operating tension was set based on the recommendation that the operating tension of PET film should be set to a 6.67% yield strength or below [51]. The drying temperature was set so that thermal deformation of the material could be observed within the appropriate temperature range of the PET film. Therefore, by setting the dryer temperature to 100 °C, the MD and CMD temperature distribution of the material and resulting strain distribution were predicted from the operating speed and operating tension. To verify the predicted temperature distribution of the material based on the CFD analysis, the experiment was conducted using the roll-to-roll manufacturing system shown in Figure 2. To measure the material temperature, a data acquisition device (GP10, Yokogawa, Tokyo, Japan), shown in Figure 5a, was used, in which the device was connected to thermocouples to record the temperature. Before the material entered the dryer inlet, five thermocouples were attached to the PET film at constant intervals in the CMD (see Figure 5b) to transport the web.

We deduced a web strain profile by using the estimated web temperature distribution results as the boundary conditions of a structural analysis model. To accurately predict the elastic and thermal strains, the elastic modulus in Figure 3a and coefficient of thermal expansion in Figure 3b were measured according to the temperature and applied. The PET film experimented on in this study generally has a decreasing elastic modulus as the temperature increases, whereas the coefficient of thermal expansion increases to a certain temperature before decreasing. Accordingly, the elastic strain increased with the temperature of the PET film, while the thermal strain increased until 80 °C and then decreased at temperatures higher than 80 °C under the operating tension condition.

## 3. Results and Discussion

**Figure 6:**
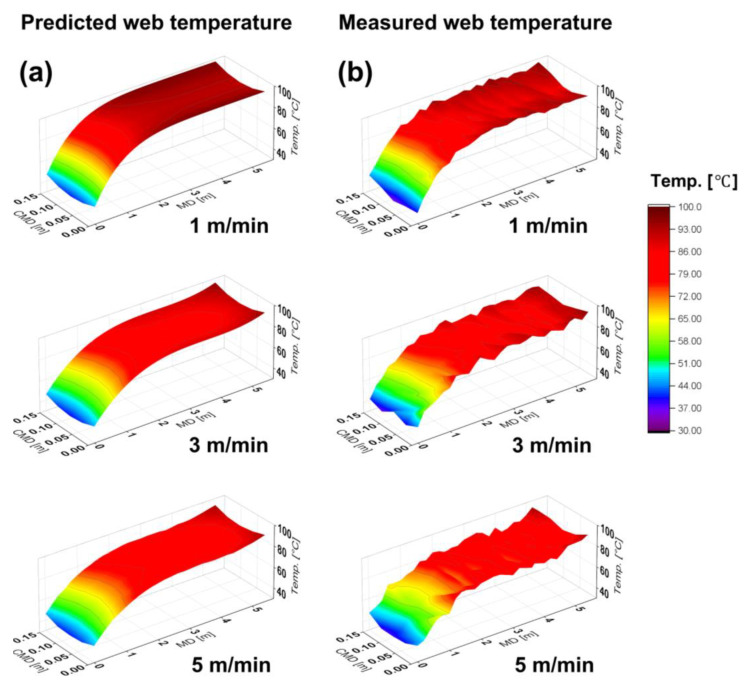
Web temperature in dryer depending on operating speed: (**a**) predicted web temperature with computational fluid dynamics analysis; (**b**) measured web temperature through roll-to-roll web transporting experiment.

The temperature distribution inside the dryer and the PET material temperature distribution were predicted based on the CFD analysis. Figure 6a illustrates the predicted PET temperature distribution when the operating speeds are 1 m/min, 3 m/min, and 5 m/min, respectively. Because the web is simultaneously heated inside the dryer and transported due to the nature of the roll-to-roll manufacturing system, an inconsistent temperature distribution occurs on the material in the MD and CMD. In general, the temperature distribution of the material is significantly affected by the operating speed, because the flow pattern inside the dryer varies depending on the operating speed of the material, and the length of time for which the material is heated inside the dryer changes. Furthermore, the target dryer system has a plate installed in the MD underneath the hot air inlet, which ensures that the high-temperature air is not applied directly to the material; instead, the material is heated by the ambient temperature inside the dryer. Therefore, the edge of the material has a higher temperature distribution than that at the center in the CMD. When the operating speed is low, the material heating time inside the dryer increases, thus approaching the set dryer temperature at all points in the MD and CMD. Consequently, the maximum CMD temperature deviation tends to decrease.

The web temperature distribution was measured to verify the predicted material temperature distribution based on the CFD analysis. Under the operating speeds shown in Table 3, the web temperature distribution in the MD was measured at each thermocouple point, and the results are illustrated in Figure 6b. The CMD temperature deviation was deduced from the temperature data measured at each point, and the maximum CMD temperature deviation according to the operating speed is presented in Table 3. The same tendency as that of the predicted results for the web moving in the MD was exhibited, and the maximum error between the predicted temperature distribution and measured values was approximately 1.3%. It is predicted that such an error occurs because the temperature of probe attached on web does not perfectly match the temperature of web in the temperature measurement process. In addition, due to the deterioration of the dryer system, there may be errors with boundary conditions applied to the dryer CFD model.

**Figure 7:**
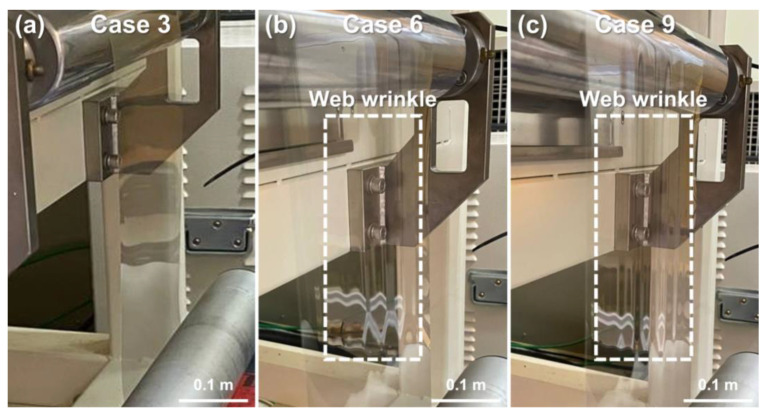
Observed wrinkles caused by strain deviation of web: (**a**) no wrinkles; (**b**) wrinkles due to increased operating speed; (**c**) severe wrinkles.

A structural analysis was performed by setting the predicted web temperature profile to the boundary conditions and applying tension through the velocity difference of the driven rolls on both sides of the web. The results of predicting the maximum strain deviation from the operating speed and operating tension are shown in Table 3. When the CMD temperature deviation increased with an increase in the operating speed, the variations in the elastic modulus and coefficient of thermal expansion of the web in the CMD increased; therefore, an increase in the maximum strain deviation was exhibited. The degree of web wrinkle defect—which may have been caused by the predicted maximum strain deviation—was experimentally observed by applying the same operating speed and operating tension in each case. After the drying section, the web was sampled on a flat plane to quantify the degree of wrinkles by measuring ZD deformation. The web form of Case 3, where the web wrinkle defect did not occur, is shown in Figure 7a. In addition, Figure 7b shows the wrinkle formed when the Case 6 operating conditions were applied; a maximum ZD deformation of approximately 2.2 mm was observed. A ZD deformation of approximately 1.4 mm was observed in Case 8, where the operating speed was 5 m/min and the operating tension was 6 kgf. Figure 7c shows Case 9—the most severe case of wrinkle defect—where the maximum ZD deformation was approximately 3.6 mm. Excluding Cases 6, 8, and 9, no other cases had a wrinkle defect large enough to observe due to strain deviation. Consequently, visually observable web wrinkle defects occur in this PET film when the maximum strain deviation caused by the CMD temperature deviation is at least 0.0000769 mm/mm. The presence of a web wrinkle defect may cause a thickness deviation in a functional layer manufactured on the web, which is directly related to the quality degradation of the final product. To decrease the occurrence of wrinkle defects, an appropriate operating speed that reduces the CMD temperature deviation must be set. The operating speed must consider the productivity and drying time of the printing or coating layers and be optimized according to the CMD temperature deviation based on the CFD analysis. Additionally, the maximum tension range must be selected by measuring the elastic modulus and thermal conductivity according to the temperature, and the operation tension must be optimized based on the maximum possible strain deviation resulting from the structural analysis that applied the predicted web temperature distribution.

## 4. Conclusions

This study performed a computer simulation and experiment for web wrinkle defects that may occur in the roll-to-roll manufacturing process. Due to the nature of the roll-to-roll manufacturing system, an inconsistent temperature distribution occurs on the material in the MD and CMD when the web is simultaneously heated and transported inside the dryer. In general, the flow pattern inside the dryer changes according to the operating speed, and the heating time of the web inside the dryer also varies; thus, the material temperature distribution is substantially affected by the operating speed. Because the elastic modulus and thermal conductivity of the web vary depending on the temperature, strain deviation occurs at different parts of the tensioned web. Therefore, strain deviation increases when the operating tension increases under an uneven temperature distribution, which was confirmed to cause ZD deformation. When the web temperature was measured using thermocouples, a maximum CMD temperature deviation of 3.8 °C was generated on the PET film inside the dryer system, and the web wrinkle defect was visually observed when a strain deviation of at least 0.0000769 mm/mm occurred. The presence of a web wrinkle defect can cause a thickness deviation in the functional layer manufactured on the web, thus causing difficulty in assembling the final products or reducing the performance uniformity.

The results suggest that an appropriate operating speed should be set to reduce the CMD temperature deviation and hence reduce the instances of wrinkle defects. The speed range must be set so that the minimum speed range ensures the drying time of the printing or coating layers and so that the maximum operating speed guarantees a sufficient productivity of the roll-to-roll manufacturing system. The CMD temperature deviation should be considered to optimize the operating speed. In addition, the results also imply that the maximum tension range must be set by considering the changes in the mechanical properties caused by the web temperature profile inside the dryer, and the appropriate operating tension must be selected by predicting the maximum possible strain deviation.

## Figures and Tables

**Table 1:** Properties of polyethylene terephthalate (PET) film.

Material	Property	Unit	Value
PET film	Width	mm	150
	Thickness	mm	0.1
	Density	kg/m^3^	1390
	Elastic modulus (25 ℃)	MPa	2510
	Poisson’s ratio	-	0.33
	Yield strength	MPa	110
	Specific heat	J/kg∙K	1172
	Thermal conductivity	W/m∙K	0.256
	Coefficient of thermal expansion (25 ℃)	${℃}^{-1}$	0.00003

**Table 2:** Computational Fluid Dynamics (CFD) dryer model specification and boundary conditions of dryer system.

CFD Model Specification	
Solver	Pressure-based
Dimensions	5.50 m × 0.64 m × 1.28 m
Number of nodes	15,981,533
Average mesh skewness	0.22
Moving web	Solid motion
**Boundary Conditions**	
Heat source	Hot air, infrared
Hot air temperature	100.0 ℃
Hot air inlet speed	0.05 m/s
Hot air outlet pressure	Negative pressure
Wall	No-slip

**Table 3:** Results of measured web temperature and estimated strain distribution for Cases 1–9.

Case	Operating Speed [m/min]	Operating Tension [kgf]	Max. Predicted CMD Temperature Deviation [℃]	Max. Measured CMD Temperature Deviation [℃]	Max. Strain Deviation $\left(\times 10^{-5}\right)$ [mm/mm]
1	1	2	1.9	2.1	1.49
2		6			3.63
3		10			6.53
4	3	2	2.5	2.8	2.02
5		6			5.40
6		10			9.01
7	5	2	3.5	3.8	3.13
8		6			7.69
9		10			13.11

## Data Availability

The data presented in this study are available from the corresponding author upon reasonable request.
